# Parent and family impact of raising a child with perinatal stroke

**DOI:** 10.1186/1471-2431-14-182

**Published:** 2014-07-14

**Authors:** Taryn B Bemister, Brian L Brooks, Richard H Dyck, Adam Kirton

**Affiliations:** 1 Department of Psychology, University of Calgary, 2500 University Drive NW, Calgary, AB T2N 1N4, Canada; 2 Calgary Pediatric Stroke Program, Room C1-320, Alberta Children’s Hospital, 2888 Shaganappi Trail NW, Calgary, AB T3B 6A8, Canada; 3 Neurosciences, Brain Injury and Rehabilitation Program, Alberta Children’s Hospital, 2888 Shaganappi Trail NW, Calgary, AB T3B 6A8, Canada; 4 Departments of Paediatrics and Clinical Neurosciences, Faculty of Medicine, University of Calgary, 3330 Hospital Drive NW, Calgary, AB T2N 4N1, Canada; 5 Alberta Children’s Hospital Research Institute, Heritage Medical Research Building, Room 293, 3330 Hospital Drive NW, Calgary, AB T2N 4N1, Canada

**Keywords:** Perinatal stroke, Caregivers, Parent impact, Family impact, Pediatric neurological conditions, Pediatric disabilities, Gender differences

## Abstract

**Background:**

Perinatal stroke is a leading cause of early brain injury, cerebral palsy, and lifelong neurological morbidity. No study to date has examined the impact of raising a child with perinatal stroke on parents and families. However, a large breadth of research suggests that parents, especially mothers, may be at increased risk for psychological concerns. The primary aim of this study was to examine the impact of raising a child with perinatal stroke on mothers’ wellbeing. A secondary aim was to examine how caring for a child with perinatal stroke differentially affects mothers and fathers.

**Methods:**

In Study I, a matched case-control design was used to compare the wellbeing of mothers of children with perinatal stroke and mothers of children with typical development. In Study II, a matched case-control design was used to compare mother-father dyads. Participants completed validated measures of anxiety and depression, stress, quality of life and family functioning, marital satisfaction, and marital distress. Parents of children with perinatal stroke also completed a recently validated measure of the psychosocial impact of perinatal stroke including guilt and blame outcomes. Disease severity was categorized by parents, validated by the Pediatric Stroke Outcome Measure (PSOM), and compared across the above outcomes in Study I.

**Results:**

A total of 112 mothers participated in Study I (*n* = 56 per group; mean child age = 7.42 years), and 56 parents participated in Study II (*n* = 28 per group; mean child age = 8.25 years). In Study I, parent assessment of disease severity was correlated with PSOM scores (γ = 0.75, *p* < .001) and associated with parent outcomes. Mothers of children with mild conditions were indistinguishable from controls on the outcome measures. However, mothers of children with moderate/severe conditions had poorer outcomes on measures of depression, marital satisfaction, quality of life, and family functioning. In Study II, mothers and fathers had similar outcomes except mothers demonstrated a greater burden of guilt and higher levels of anxiety.

**Conclusions:**

Although most mothers of children with perinatal stroke adapt well, mothers of children with moderate/severe conditions appear to be at higher risk for psychological concerns.

## Background

Ischemic perinatal stroke is a focal interruption of blood supply in the brain that is caused by the blockage of a blood vessel between 20 weeks of fetal life to 28 days of life [1]. This cardiovascular event occurs in at least 1 in 2500 live births and is a leading cause of lifelong neurological disability. The majority of perinatal stroke survivors experience chronic motor impairments, while other typical outcomes include seizures, cognitive deficits, sensorimotor deficits, and behaviour problems [2]. This condition impacts the child, parents, and family across complex aspects of life and over the child’s lifespan. Despite this, no study to date has examined the wellbeing of parents of children with perinatal stroke.

Several studies have examined the wellbeing of mothers of children with chronic neurological conditions, such as cerebral palsy, epilepsy, and developmental disabilities. These studies suggest that, although many mothers of children with perinatal stroke will adapt well, they may be at elevated risk for psychological concerns. Heightened rates of stress and depression have consistently been found among parents of children with cerebral palsy, while heightened rates of anxiety have been found in response to acute stressors (e.g., child’s diagnosis) [3,4]. Similar findings have emerged in the epilepsy and developmental disability literature with meta-analyses and reviews supporting affected mothers’ increased susceptibility to stress, depression, and other mental health concerns [5-7]. Even though these mothers tend to have an increased risk for psychological concerns, it is important to note that a large portion of them demonstrate resiliency [8].

Caregivers’ quality of life is influenced by their psychological functioning as well as other aspects of wellbeing (health, independence, relationships, beliefs, and environment [9]). In line with previous research, mothers of children with cerebral palsy and other neurological disabilities tend to report poorer quality of life than mothers of typically developing children [3,9,10]. An extensive review of 46 studies on mothers of children with cerebral palsy highlights the consistency of this finding within the literature [3] with only two studies failing to find such an effect [9,11]. Nonetheless, many of these mothers continue to report quality of life within the normal range.

Fewer studies have focused on the paternal impact of raising a child with a neurological disability [12]. The studies that have included fathers have generally found them to have similar or better psychological outcomes than mothers [12,13]. A meta-analysis of 229 adult caregiver studies found that male caregivers tend to report lower levels of stress and depression in conjunction with higher levels of wellbeing and physical health than female caregivers, although the effects were small to very small [14]. The authors note that these gender differences may stem from females’ increased caregiver responsibilities and stressors. Furthermore, other studies have observed gender differences in the ways that parents perceive and cope with stress [15].

Although there is an emphasis on primary caregivers in pediatric disability research, a family systems perspective is increasingly being employed with an emphasis on parental, marital, and family functioning. In terms of marital functioning, research indicates that there is an elevated risk of divorce and separation among parents of children with disabilities, albeit the effect is smaller than previously believed (i.e., 3-7% increased risk [16]). Hence, many parents of children with disabilities have marriages within the normal range of function and dysfunction [17]. Some authors still insist that parents of children with disabilities have lower marriage quality and lower marital satisfaction [18,19]. Alternatively, some authors argue that the challenge of coping with a child’s disability can strengthen and enrich an already satisfying marriage [20].

With respect to family functioning, the results in the literature have largely been mixed. More problematic family functioning has been observed in families with children with disabilities [21,22], while other studies have failed to find such an effect [11,23]. In light of these findings, Coffey suggests that caring for a child with a disability may strain the family system by restricting family activities, but it also may strengthen the family system by bonding family members [24]. Regardless, there is widespread recognition of the value of family functioning and its effect on individual family members.

Despite the overall impact of pediatric disabilities on parents’ wellbeing, variation in outcomes exists depending on the type and severity of the child’s condition [25]. For instance, condition-specific effects have been observed for epilepsy, cerebral palsy, and pervasive developmental disorder [10,26,27]. The differences in outcomes have been attributed to the conditions’ unique presentations and associated challenges and strengths. Because no studies to date have evaluated the wellbeing of parents of children with perinatal stroke, the specific impact of this condition is yet to be determined. As noted by Bemister and colleagues [28], these parents may present with elevated levels of guilt and blame compared to other neurological disabilities. This may occur because parents are aware of the timing of their child’s stroke, but they are unaware of a definitive cause; as a result, they may make causal attributions involving apparent events around the time of the stroke (e.g., their actions during the last trimester and/or medical staff actions during delivery).

In addition, differences in caregiver wellbeing have emerged within specific conditions dependent on child, parent, and environmental factors (e.g., child behaviour problems, parent self-esteem, and socioeconomic status [25]). One commonly researched determinant is condition severity. Even though there are inconsistent findings on this topic, milder conditions have been associated with better outcomes for parents of children with cerebral palsy [25,27]. These results may be due to the relative reduction of caregiver demands.

The existing literature highlights the importance of examining the maternal, parental, and familial impact of raising a child with perinatal stroke. Many families affected by perinatal stroke remain underserved in our clinical experience, which may be partially due to the paucity of research on this population. Family-based research studies on perinatal stroke may augment the existing literature, as well as enhance existing resources, supports, and services available to affected families. Furthermore, such research is consistent with family-centered care, an increasingly revered service delivery approach for pediatric neurological conditions [29].

The primary aim of this study is to examine the impact of raising a child with perinatal stroke on mothers’ wellbeing, as evident by measures of their depression symptoms, anxiety symptoms, stress levels, quality of life, marital distress, marital satisfaction, and family functioning. A secondary aim is to examine how caring for a child with perinatal stroke differentially affects mothers and fathers. Based on previous literature, it was hypothesized that mothers would have worse outcomes in all domains measured relative to mothers of children with typical development and fathers of children with perinatal stroke.

## Methods

### Participants

Mothers of children with typical development and mothers and fathers of children with perinatal stroke were identified through the Alberta Perinatal Stroke Project (APSP). APSP is a population-based research cohort of >180 perinatal stroke patients and >50 healthy controls in southern Alberta. Mothers of children with typical development were additionally recruited through a research participation system at the University of Calgary and community advertisements (printed and online). The biological parents of children 0-18 years with a clinico-radiographically confirmed perinatal stroke syndrome (neonatal arterial ischemic stroke, periventricular venous infarction, or arterial presumed perinatal stroke [30]) and the biological mothers of typically developing children 0-18 years (no known neurological or developmental conditions) were included in this study. Participants were excluded if they had less than nine years of formal education (excluding schooling prior to four years of age) or were unable to fluently read English (based on self-report).

### Procedure

Study I and II were conducted concurrently between August 2012 and June 2013 as part of an ongoing research project, and ethics approval was obtained from the Conjoint Health Research Ethics Board at the University of Calgary. Parents were explained the study via telephone or email (depending on their preference), and consent was obtained prior to sending them a link to the questionnaire battery. Individual links were sent to the parents using the online survey software, Qualtrics, which enabled participants to save and alter their responses prior to submission. All participants were given the option to complete paper versions of the questionnaires. The vast majority of the participants received a $10 eGift card in recognition of their contribution, while the participants recruited through the university received one bonus credit toward a course. The data were downloaded from Qualtrics and stored in a secure database at the Alberta Children’s Hospital.

### Measures

#### Demographics

The Demographics Questionnaire is a 26-item scale created for an ongoing research project [28] to assess relevant background information about the participants, including their age, income, education, and ethnicity (see Additional file 1 for the scale). This questionnaire has not yet been validated.

#### Anxiety and depression

The Hospital Anxiety and Depression Scale (HADS) is a 14-item scale that measures self-reported symptoms of anxiety (HADS-A) and depression (HADS-D) within the past week [31]. Comprehensive reviews of the HADS suggest it has good reliability and validity in hospital and community populations [32]. Furthermore, the scale is commonly used among parents of children with and without chronic conditions.

#### Perceived stress

The Perceived Stress Scale (PSS) is a 14-item scale that measures the extent to which situations are judged as being stressful, uncontrollable, unpredictable, and overloading [33]. The PSS has good to very good reliability and validity, and it has been deemed an effective tool for evaluating stress in parents of children with disabilities [34].

#### Family functioning and quality of life

The Pediatric Quality of Life Inventory Family Impact Module (PedsQL FIM) is a 36-item scale that measures the impact of pediatric health conditions on parent quality of life and family functioning [35]. The PedsQL FIM generates three scores – Parents’ Health-Related Quality of Life (HRQL), Family Functioning, and Total Score – all of which have demonstrated internal consistency and construct validity [36]. The PedsQL FIM has been widely used among parents of children with chronic conditions, but it is also suitable for healthy controls.

#### Family impact

The APSP Parental Outcome Measure (POM) is a 26-item scale that measures the impact of perinatal stroke on parents and families [28]. As such, this scale was not administered to parents of children with typical development. The POM has three subscales that measure parents’ psychosocial impact, guilt, and blame. Evidence for the POM’s reliability and validity was gathered in its original validation study with parents of children with perinatal stroke.

#### Marital strain

Only participants in marital or common-law relationships completed the following scales:

The Dyadic Adjustment Scale (DAS) is a 32-item scale assessing distress in marital or common-law relationships [37]. The DAS is one of the most established questionnaires of its kind, and it has been shown to be theoretically-based, valid, and reliable [37,38].

The Kansas Marital Satisfaction Scale (KMSS) is a global measure of marital satisfaction that was administered to complement the DAS [39,40]. The KMSS is psychometrically sound and consists of three items that assess satisfaction with one’s partner, marriage, and relationship [41].

### Study part I: perinatal stroke vs. typical development

#### Statistical analyses

Descriptive statistics for demographic variables were calculated and comparisons were made between the mothers of children with perinatal stroke and the mothers of children with typical development using chi-square analyses for categorical data and *t*-tests for continuous data. A preliminary examination of the data was conducted using scatterplots and the results revealed substantial variation in the outcome measures among the mothers of children with perinatal stroke. As a result, the mothers were grouped according to the severity of their child’s condition: mild and moderate/severe (moderate and severe conditions were collapsed together due to the small sample size of severe cases; see Results for details of this process). Nonparametric statistics were conducted for the rest of the analyses due to the unequal sample sizes and heterogeneity of variance among the outcome measures. Specifically, Kruskal-Wallis H tests were used to compare the groups on the outcome measures, followed up with Mann-Whitney *U* tests. Bonferroni adjustments were applied to correct for family-wise error rates, and all statistics were conducted with IBM SPSS Statistics for Windows Version 20.0.

## Results

### Sample

A total of 82 mothers of children with perinatal stroke met the study’s inclusion criteria and were recruited as part of a larger ongoing research study [28]. A total of 62 mothers of children with typical development met the study’s inclusion criteria and were recruited from community advertisements (*n* = 34), the university (*n* = 15), and the APSP control database (*n* = 13). Among them, 56 were successfully matched to mothers of children with perinatal stroke based on their child’s sex, age (±2 years), and total gross family income (±1 category). As highlighted in Table 1, the mothers of children with perinatal stroke were comparable to the mothers of children with typical development on all of the demographic variables examined.

**Table 1:** Demographics as a percentage of the sample: perinatal stroke vs. typical development

	**Perinatal stroke**	**Typical development**	**Statistical value**
**Child demographics**	***n *** **(%)**	***n *** **(%)**	**(*p*-value)**
Age of child (years)	Mean = 7.34 (SD = 5.20),	Mean = 7.49 (SD = 5.15),	-0.15 (.88)
	Range = 0.75-18	Range = 0.50-18	
Child’s sex			
Male	29 (51.79%)	29 (51.79%)	
Female	27 (48.21%)	27 (48.21%)	
Ethnicity			1.46 (.23)
Caucasian/White	48 (85.71%)	43 (76.79%)	
Other	8 (14.29%)	13 (23.21%)	
PSOM total^a^	2.28 (2.43), 0-10	--	--
Severity of condition^b^			--
Mild	29 (51.8%)	--	
Moderate	19 (34.0%)	--	
Severe	8 (14.2%)	--	
**Parent demographics**			
Age of parents (years)	Mean = 38.05 (SD = 6.64), Range = 27-55	Mean = 37.82 (SD = 7.23), Range = 22-51	.18 (.86)
Caregiver status			2.05 (.36)
Lone caregiver	8 (14.29%)	11 (19.64%)	
Co-caregiver	48 (85.71%)	45 (80.36%)	
Mental health concerns prior to child’s birth			.73 (.39)
Yes	13 (23.21%)	17 (30.36%)	
No	43 (76.79%)	39 (69.64%)	
Total gross household income (CDN)			2.02 (.37)
< $70,000	19 (33.93%)	25 (44.64%)	
$71,000-110,000	18 (32.14%)	12 (21.43%)	
> $111,000	19 (33.93%)	19 (33.93%)	
Hours spent working outside of the home			3.07 (.55)
<10	25 (44.64%)	18 (32.14%)	
10-30	14 (25.0%)	15 (26.79%)	
> 30	17 (30.36%)	23 (41.07%)	
Education level			4.41 (.35)
≤ High school certificate	15 (26.79%)	10 (17.86%)	
College certificate or diploma	20 (35.71%)	14 (25.0%)	
Bachelor’s degree	14 (25.0%)	21 (37.5%)	
Master’s, doctorate or professional degree	7 (12.5%)	11 (19.64%)	

*Note. n* =56 for both groups. All statistical values are *X*^2^ unless otherwise specified.

^a^Statistical value is a *t*-value. ^b^*n* = 49. ^c^Rating is based on parents’ self-reported perceptions of the severity of their child’s condition.

Mothers of children with perinatal stroke were divided into mild (*n* = 29) and moderate/severe (*n* = 27) conditions based on parent classifications. These classifications were in very strong agreement with the results of the standardized Pediatric Stroke Outcome Measure (PSOM; [42]), which was available for 49 of the 56 cases (Goodman and Krusk’s gamma correlation (γ) = 0.75, *p* < .001). These groups did not differ on any of the demographic variables described in Table 1 (data not shown).

The mild, moderate/severe, and typical development conditions were compared on the outcome variables, the results of which are summarized in Table 2. Pairwise comparisons with Bonferroni corrections were conducted on all significant findings and are listed in Table 3. The mothers of children with typical development recruited from different sources were also compared on the outcome variables, but no statistically significant differences emerged (data not shown).

**Table 2:** Comparison of mothers of children with typical development, mild conditions, and moderate/severe conditions on outcome variables

	**Median [95% CI]**				
	**Typical dev. condition**	**Mild condition**	**Moderate/ severe condition**	***X***^***2 ***^**(*p*-value)**	**Effect size (*η***^**2**^**)**
**Anxiety & depression**					
HADS-A	7.00 [5.50-8.00]	7.00 [4.00-8.00]	8.00 [5.00-10.00]	2.11 (.35)	.02
HADS-D	3.00 [2.00-4.00]	2.00 [1.00-3.00]	5.00 [4.00-9.00]	12.43 (.002)*	.11
**Perceived stress**					
PSS	22.50 [19.00-25.00]	21.00 [15.51-24.00]	26.00 [20.00-30.00]	4.93 (.08)	.04
**Marital strain**					
KMSS^a,b^	18.00 [17.00-18.00]	18.00 [17.00-21.00]	15.00 [12.00-18.00]	8.12 (.017)*	.09
DAS^a,b^	115.00 [106.01-120.00]	113.00 [105.00-122.00]	105.00 [88.00-116.00]	2.76 (.25)	.05
**Parent & family adaptation**					
PedsQL FIM^a^					
Total^a^	78.13 [70.83-85.42]	79.86 [71.53-88.19]	53.47 [38.89-58.33]	24.38 (<.001)*	.22
Parent HRQL^a^	72.50 [67.50-80.00]	81.25 [72.50-90.00]	60.00 [49.37-65.00]	12.08 (.002)*	.11
Family Functioning^a^	84.38 [70.31-90.63]	87.50 [65.63-100.00]	46.87 [34.38-62.42]	25.77 (<.001)*	.23

*Note. n* = 56 for typical development, *n* = 29 for mild condition, and *n* = 27 for moderate/severe condition. Higher scores indicate poorer functioning unless specified otherwise. A *η*^2^ of .01 is a small effect, .06 is a medium effect, and .14 is a large effect [50].

*p-value is significant correcting for family-wise error rate (*p* < .025 for HADS, *p* < .025 for measures of marital strain, and *p* < .017 for PedsQL FIM).

^a^Higher scores indicate better functioning. ^b^*n* = 45 for typical development, *n* = 27 for mild, and *n* = 24 for moderate/severe.

**Table 3:** Pairwise comparisons on outcome variables

	**Mann-Whitney *U *** **(*p *-value)**	**Effect size (*r*)**
**HADS-D**		
Typical dev. vs. mild	644.50 (.12)	-.17
Typical dev. vs. moderate/severe	500.50 (.01)*	-.27
Mild vs. moderate/severe	183.50 (.001)*	-.46
**PedsQL FIM total**		
Typical dev. vs. mild	784.50 (.80)	-.03
Typical dev. vs. moderate/severe	283.50 (<.001)*	-.50
Mild vs. moderate/severe	139.00 (<.001)*	-.55
**PedsQL parent HRQL**		
Typical dev. vs. mild	748.50 (.56)	-.06
Typical dev. vs. moderate/severe	459.00 (.004)*	-.32
Mild vs. moderate/severe	188.00 (.001)*	-.45
**PedsQL family functioning**		
Typical dev. vs. mild	727.50 (.43)	.08
Typical dev. vs. moderate/severe	305.50 (<.001)*	-.23
Mild vs. moderate/severe	111.50 (<.001)*	-.62
**KMSS**^**a**^		
Typical dev. vs. mild	544.50 (.45)	-.09
Typical dev. vs. moderate/severe	377.00 (.04)	-.25
Mild vs. moderate/severe	171.00 (.003)*	-.41

*Note. n* = 56 for typical development, *n* = 29 for mild condition, and *n* = 27 for moderate/severe condition. A *r* of |.1| is a small effect, |.3| is a medium effect, and |.5| is a large effect [51].

*p-value (one-way) < .017.

^a^*n* = 45 for typical development, *n* = 27 for mild, and *n* = 24 for moderate/severe.

### Anxiety and depression

Although no statistical difference was found in symptoms of anxiety among the conditions (HADS-A; *p* = .35), a statistically significant difference emerged when examining symptoms of depression (HADS-D; *p* = .002). Pairwise comparisons revealed that the moderate/severe condition (*Mdn* = 5.00) had significantly more symptoms of depression than the mild condition (*Mdn* = 2.00, *p* = .001) and typical development condition (*Mdn* = 3.00, *p* = .01). However, no statistical difference was found between the mild and typical development conditions (*p* = .12).

### Perceived stress

A similar pattern was observed in perceived stress among the three conditions, but the results did not reach statistical significance (PSS; *p* = .08).

### Family functioning and quality of life

Significant differences were found among the groups in the PedsQL FIM Total score (*p* < .001), Family Functioning score (*p* < .001), and Parent Health-Related Quality of Life score (HRQL; *p* = .002). Pairwise comparisons showed that the moderate/severe condition (Total *Mdn* = 53.47; Family Functioning *Mdn* = 46.87; HRQL *Mdn* = 60.00) had significantly lower scores (worse functioning) than the mild condition (Total *Mdn* = 79.86, *p* < .001; Family Functioning *Mdn* = 87.50, *p* < .001; Parent HRQL *Mdn* = 81.25, *p* < .001) and the typical development condition (Total *Mdn* = 78.13, *p* < .001; Family Functioning *Mdn* = 84.38, *p* < .001; Parent HRQL *Mdn* = 72.50, *p* = .004) on all three outcomes. No statistical differences existed between the mild and typical development conditions on the outcomes (*p* = .80 for Total; *p* = .43 for Family Functioning; and *p* = .56 for HRQL).

### Marital distress and satisfaction

For both measures of marital distress (DAS) and satisfaction (KMSS), the moderate/severe condition tended to have worse outcomes. However, a statistically significant difference was only present for KMSS (*p* = .017; DAS: *p* = .25). Pairwise comparisons confirmed that the moderate/severe condition (*Mdn* = 15.00) had significantly less marital satisfaction than the mild condition (*Mdn* = 18.00; *p* = .003). No statistical differences were found between the typical development condition (*Mdn* = 18.00) and the mild condition (*p* = .45) or the moderate/severe condition (*p* = .04) after correcting the *p*-value for family-wise error rates (*p* < .017).

### Study part II: mothers vs. fathers

#### Statistical analyses

Mothers and fathers of children with perinatal stroke were compared on demographic variables using chi-square analyses for categorical data and paired samples *t*-tests for continuous data. For the primary outcome variables, the data were not normally distributed, so Wilcoxon matched pairs signed-rank tests were used throughout.

## Results

### Sample

A total of 56 parents (28 mother-father couples) of children with perinatal stroke participated in this study. The vast majority of the sample was Caucasian (92.86%) and caring for a child with a mild condition (75%). No statistical differences were found between the mothers and fathers on the demographic variables examined with the exception that fathers spent more hours working outside the home compared to mothers (*χ*^2^(4,56) = 24.83, *p* < .001; Table 4).

**Table 4:** Demographics as a percentage of the sample: mothers vs. fathers

	**Mothers**	**Fathers**	**Statistical value**
**Child demographics**	***n *** **(%)**	***n *** **(%)**	**(*p*-value)**
Child’s age (years)	Mean = 8.25 (SD = 5.82),	Mean = 8.25 (SD = 5.82),	--
	Range = 0.5-17	Range = 0.5-17	
Child’s sex			--
Male	15 (53.57%)	15 (53.57%)	
Female	13 (46.43%)	13 (46.43%)	
Ethnicity			--
Caucasian/White	26 (92.86%)	26 (92.86%)	
Other	2 (7.14%)	2 (7.14%)	
PSOM total^a^	Mean = 1.46 (SD = 1.35), Range = 0-5	Mean = 1.46 (SD = 1.35), Range = 0-5	--
Severity of condition^b^			
Mild	21 (75.00%)	20 (71.43%)	.10 (.75)
Moderate	7 (25.00%)	8 (28.57%)	
Severe	0 (0%)	0 (0%)	
**Parent demographics**			
Age (years)	Mean = 40.57 (SD = 7.87),	Mean = 42.32 (SD = 7.47),	-.06 (.95)^c^
	Range = 29-57	Range = 31-59	
Ethnicity			--
Caucasian/White	26 (92.86%)	26 (92.86%)	
Other	2 (7.14%)	2 (7.14%)	
Mental health concerns prior to child’s birth			1.02 (.50)
Yes	7 (25.00%)	4 (14.29%)	
No	21 (75.00%)	24 (85.71%)	
Total gross household income (CDN)^d^			2.00 (.37)
< $70,000	7 (25.00%)	6 (21.43%)	
$71,000-110,000	10 (35.71%)	6 (21.43%)	
> $111,000	11 (39.29%)	16 (57.14%)	
Hours spent working outside of the home			24.83 (<.001)*
<10	13 (46.43%)	2 (7.14%)	
10-30	6 (21.43%)	2 (7.14%)	
>30	9 (32.14%)	24 (85.72%)	
Education level			3.71 (.45)
≤High school certificate	4 (14.29%)	6 (21.43%)	
College certificate or diploma	7 (25.00%)	7 (25.00%)	
Bachelor’s degree	14 (50.00%)	8 (28.57%)	
Master’s, doctorate, or professional degree	3 (10.71%)	7 (25.00%)	

*Note. n* = 28 for both groups. All statistical values are *X*^2^ unless otherwise specified.

^a^*n* = 54. ^b^Rating is based on parents’ self-reported perceptions of the severity of their child’s condition. ^c^Statistical value is a *t*-value. ^d^The mothers and fathers are from the same household, and therefore the differences reported in gross family income reflect differences in perception or understanding.

*p-value < .05.

### Psychosocial outcomes

A series of Wilcoxon matched pairs signed-rank tests revealed that the mothers and fathers did not differ significantly on the majority of the outcome measures after controlling for family-wise error rates (Table 5). The only statistically significant differences were on the measures of anxiety and guilt. The results suggest that mothers have higher levels of anxiety (HADS-A: mother *Mdn* = 7.00, father *Mdn* = 5.00; Z = -1.99, *p* = .023), as well as higher levels of guilt regarding the cause of their child’s condition (POM Guilt: mother *Mdn* = 7.00, father *Mdn* = 4.00; Z = -2.33, *p* = .01) in comparison to fathers.

**Table 5:** Comparison of mothers and fathers of children with perinatal stroke on outcome variables

	**Median [95% CI]**			
	**Mothers**	**Fathers**	***Z *** **(*p*-value)**	**Effect size (*r*)**
**Anxiety & depression**				
HADS-A	7.00 [6.00-8.00]	5.00 [3.50-8.00]	-1.99 (.023)*	-.27
HADS-D	2.00 [2.00-4.49]	2.00 [1.00-5.00]	-1.14 (.13)	-.15
**Perceived stress**				
PSS	22.50 [18.00-24.00]	20.00 [16.00-26.50]	-1.11 (.45)	-.15
**Marital strain**				
KMSS^a^	18.00 [17.00-19.00]	18.00 [16.00-19.00]	-0.75 (.23)	-.10
DAS^a^	112.50 [105.00-121.00]	112.00 [104.00-127.00]	-0.20 (.42)	-.03
**Parent & family adaptation**				
PedsQL FIM^a^				
Total^a^	71.87 [59.37-84.72]	79.86 [70.84-86.11]	-.80 (.21)	-.11
Parent HRQL^a^	70.00 [60.00-90.00]	81.25 [74.00-90.00]	-1.09 (.14)	-.15
Family Functioning^a^	71.88 [56.25-89.06]	75.00 [65.62-90.63]	-0.16 (.44)	-.02
POM				
Total	34.50 [27.50-47.00]	28.00 [19.00-36.49]	-1.59 (.06)	-.21
Psychosocial Impact	20.50 [16.50-29.50]	18.50 [13.00-24.00]	-1.17 (.12)	-.16
Guilt	7.00 [4.00-9.00]	4.00 [1.50-5.00]	-2.33 (.01)*	-.31
Blame	5.50 [4.00-7.50]	6.00 [4.00-8.49]	-0.37 (.36)	-.05

*n* = 28 for each group. Higher scores indicate poorer functioning unless specified otherwise. A *r* of |.1| is a small effect, |.3| is a medium effect, and |.5| is a large effect [51].

*p-value (one-way) is significant correcting for family-wise error rate (*p* < .025 for HADS, *p* < .025 for measures of marital strain, *p* < .017 for PedsQL FIM, and *p* < .0125 for POM).

^a^Higher scores indicate better functioning.

Although no significant findings emerged for the remaining outcome variables, an examination of the effect sizes suggests that mothers may have slightly worse functioning than fathers on measures of depression, stress, quality of life, parent impact, and psychosocial functioning (Table 5). However, no differences were observed between mothers’ and fathers’ reports of marital distress and satisfaction.

## Discussion

The purpose of this study was to compare mothers of children with perinatal stroke with 1) mothers of children with typical development and 2) fathers of children with perinatal stroke. Comparisons with the typical development group revealed a promising finding: most parents of children with perinatal stroke adapt extremely well. More specifically, the mothers of children with mild conditions were indistinguishable from the control group in all of the examined outcomes (i.e., anxiety, depression, perceived stress, marital strain and satisfaction, health-related quality of life, and family functioning). Although variation in outcomes was present among the mothers of children with moderate/severe conditions, these mothers tended to have increased symptoms of depression, decreased marital satisfaction, poorer health-related quality of life, and poorer family functioning. This finding is consistent with pediatric disability research, which supports that these parents may be in need of additional resources and services [5-7,43].

Comparisons of mothers and fathers of children with perinatal stroke revealed that mothers have similar or slightly worse functioning than fathers on the outcome variables examined. The only statistically significant differences between the groups were in measures of guilt and anxiety. Mothers tended to have a greater burden of guilt regarding the cause of their child’s condition, which is likely due, at least in part, to their exceptionally intimate involvement with their child at the time of the stroke (in utero or during birth). Similarly, mothers tended to have increased levels of anxiety, which is in line with previous research on pediatric disabilities [12,13], as well as the general caregiver literature [14]. This finding is also consistent with the small, but not significantly different, gender effects observed in depression, stress, quality of life, parent impact, and psychosocial functioning – all of which suggest fathers have better outcomes. These effects may have failed to reach statistical significance due to the limited sample of fathers in the current study. Underrepresentation of fathers in caregiver research is a longstanding issue with recognized barriers involving perceived gender roles, restrictions due to employment, and fathers’ limited involvement with health professionals [44]. Swallow and colleagues provide several suggestions to help address the underrepresentation of fathers in caregiver research [44].

Data from this study build upon the existing disability literature in several ways. Foremost, this is the first study known to the authors that examines the impact of raising a child with perinatal stroke. In order to gather a preliminary and broad understanding of the parent and family impact, a case-control study design and survey methodology was utilized. This study design and methodology enabled the authors to assess seven psychosocial constructs in over 135 participants while largely controlling for demographic variables. In addition, the results of this study elicit clinically relevant questions that lay the foundation for future research studies on perinatal stroke. For instance, future research may evaluate the percentage of parents that meet criteria for psychiatric diagnoses, the impact of parent outcomes on children, and the trajectory of parents’ psychosocial functioning as the child progresses through different stages of life.

Based on the results of this study, the family impact of perinatal stroke appears to differ from other pediatric conditions in the preponderance of condition severity on parent and family outcomes. This may be because the participants were recruited from a population-based sample and the consequences of the perinatal stroke varied vastly from neurological normalcy to quadriplegia. In order to fully comprehend how perinatal stroke differs and resembles other pediatric conditions in terms of its family impact, research with chronic disease controls is required.

### Limitations

The results of this study must be interpreted within the scope of its limitations. One of the greatest limitations is that condition severity was determined based on mothers’ ratings. Hence, we are unable to eliminate the possibility that mothers’ psychosocial functioning impacted their perceptions of their child’s condition. However, an objective measure of functional impairment (i.e., PSOM) was available for 87.5% of the cases, and the results of the PSOM were in strong agreement with parent ratings. Because PSOM scores were not available for all of the participants in the study, they unfortunately could not be used as the primary measure of condition severity, and instead they were used to validate parent ratings.

Another limitation is the generalizability of the findings. The study sample consisted predominantly of educated mothers of Caucasian descent with gross family incomes of over $70,000 CDN (*Mdn* in Alberta = $89,830; Canada = $72,240 [45]). Previous research has shown that socioeconomic status and ethnic minority status are possible predictors of poor coping following the diagnosis of a pediatric disability [46]. As such, the results of this study may underestimate the overall effect of caring for a child with perinatal stroke, and they cannot be generalized to families with different demographic profiles. Future research is needed to assess the family impact of perinatal stroke among more diverse populations, including in regions beyond southern Alberta.

Lastly, this study utilized a population-based sample and included parents of children with a wide range of ages (0.5 to 18 years). Consistent with the study’s intent, this provided an overarching picture of the psychosocial effects of raising a child with perinatal stroke. However, several questions remain about the parental effects across the child’s lifespan. For example, parental distress is expected to increase in response to initial diagnoses, as well as in response to realized losses of developmental milestones and other triggers for parental recognition of childhood disability [3,47]. Longitudinal studies would help elucidate this trajectory for parents of children with perinatal stroke and the periods in which they have the highest risk for psychological concerns.

## Conclusions

The results of this study may be used to advocate for families affected by moderate/severe perinatal stroke, as well as to expand and enhance existing resources. There is increasing recognition of perinatal or pediatric stroke as a unique neurological condition that merits specialized clinics and services. Similarly, family supports tailored for this specific population have emerged in the past decade (e.g., the APSP Parent Support Group). The results of this study may inform such supports and services by identifying parents at higher risk for psychological concerns (parents of children with moderate/severe conditions), as well as identifying areas of concern (parent depression, marital satisfaction, health-related quality of life, and family functioning). Family-based supports are not only beneficial for the parents, but also the entire family system [48]. Parent wellbeing has been consistently shown to positively influence the health and psychosocial functioning of children with pediatric disabilities [5,6,49]. Thus, the results of this study may be utilized by clinicians, policymakers, and researchers to help enhance the quality of life of parents, families, and children affected by perinatal stroke.

## Abbreviations

APSP: Alberta Perinatal Stroke Project; AIHS: Alberta Innovates – Health Solution; CDN: Canadian; CIHR: Canadian Institutes of Health Research; DAS: Dyadic Adjustment Scale; HADS: Hospital Anxiety and Depression Scale (-A: -Anxiety; -D: Depression); HRQL: Health-Related Quality of Life; KMSS: Kansas Marital Satisfaction Scale; Mdn: Median; PedsQL FIM: Pediatric Quality of Life Inventory Family Impact Module; POM: (Alberta Perinatal Stroke Project) Parental Outcome Measure; PSOM: Pediatric Stroke Outcome Measure; PSS: Perceived Stress Scale.

## Competing interests

BB receives funding from a test publisher (Psychological Assessment Resources, Inc.). No competing interests exist for TB, RD, and AK.

## Authors’ contributions

TB was responsible for all aspects of the study. BB, AK, and RD provided guidance to TB, contributed to the study design, and extensively reviewed the manuscript. All authors read and approved the final manuscript.

## Pre-publication history

The pre-publication history for this paper can be accessed here:

http://www.biomedcentral.com/1471-2431/14/182/prepub

## Supplementary Material

Additional file 1 Demographics questionnaire: parents of children with perinatal stroke. Click here for file

## References

[B1] Raju TNK Nelson KB Ferriero D Lynch JK Ischemic perinatal stroke: summary of a workshop sponsored by the National Institute of Child Health and Human Development and the National Institute of Neurological Disorders and Stroke Pediatrics 2007 120 3 609 616 17766535 10.1542/peds.2007-0336

[B2] Golomb MR Outcomes of perinatal arterial ischemic stroke and cerebral sinovenous thrombosis Semin Fetal Neonatal Med 2009 14 5 318 322 19647504 10.1016/j.siny.2009.07.003

[B3] Pousada M Guillamón N Hernández-Encuentra E Muñoz E Redolar D Boixadós M Gómez-Zúñiga B Impact of caring for a child with cerebral palsy on the quality of life of parents: a systematic review of the literature J Dev Phys Disabil 2013 25 5 545 577

[B4] Guyard A Fauconnier J Mermet MA Cans C Impact on parents of cerebral palsy in children: a literature review Arch Pediatr 2011 18 2 204 214 21196101 10.1016/j.arcped.2010.11.008

[B5] Duffy LV Parental coping and childhood epilepsy: the need for future research J Neurosci Nurs 2011 43 1 29 35 21338042

[B6] Ferro MA Speechley KN Depressive symptoms among mothers of children with epilepsy: A review of prevalence, associated factors, and impact on children Epilepsia 2009 50 11 2344 2354 19694788 10.1111/j.1528-1167.2009.02276.x

[B7] Singer GHS Meta-analysis of comparative studies of depression in mothers of children with and without developmental disabilities Am J Ment Retard 2006 111 3 155 169 16597183 10.1352/0895-8017(2006)111[155:MOCSOD]2.0.CO;2

[B8] Heiman T Parents of children with disabilities: resilience, coping, and future expectations J Dev Phys Disabil 2002 14 2 159 171

[B9] Terra VC Cysneiros RM Schwartzman JS Teixeira MCTV Arida RM Cavalheiro EA Scorza FA de Albuquerque M Mothers of children with cerebral palsy with or without epilepsy: a quality of life perspective Disabil Rehabil 2011 33 5 384 388 21142651 10.3109/09638281003611052

[B10] Mugno D Ruta L D'Arrigo VG Mazzone L Impairment of quality of life in parents of children and adolescents with pervasive developmental disorder Health Qual Life Outcomes 2007 5 22 15 17466072 10.1186/1477-7525-5-22 PMC1868708

[B11] Magill-Evans J Darrah J Pain K Adkins R Kratochvil M Are families with adolescents and young adults with cerebral palsy the same as other families? Dev Med Child Neurol 2001 43 7 466 472 11463177 10.1017/s0012162201000858

[B12] Pelchat D Lefebvre H Levert M Gender differences and simililarities in the experience of parenting a child with a health problem: current state of knowledge J Child Health Care 2007 11 2 112 131 17494986 10.1177/1367493507076064

[B13] Ha J Hong J Seltzer MM Greenberg JS Age and gender differences in the well-being of midlife and aging parents with children with mental health or developmental problems: Report of a national study J Health Soc Behav 2008 49 3 301 316 18771065 10.1177/002214650804900305 PMC2836839

[B14] Pinquart M Sörensen S Gender differences in caregiver stressors, social resources, and health: an updated meta-analysis J Gerontol B Psychol Sci Soc Sci 2006 61 1 33 45 10.1093/geronb/61.1.p33 16399940

[B15] Matud MP Gender differences in stress and coping styles Pers Indiv Differ 2004 37 7 1401 1415

[B16] Risdal D Singer GHS Marital adjustment in parents of children with disabilities: a historical review and meta-analysis Res Pract Pers Sev D 2004 29 2 95 103

[B17] Sobsey D Marital stability and marital satisfaction in families of children with disabilities: chicken or egg? Dev Disabil Bull 2004 32 1 62 83

[B18] Florian V Findler L Mental health and marital adaptation among mothers of children with cerebral palsy Am J Orthopsychiatry 2001 71 3 358 367 11495338 10.1037/0002-9432.71.3.358

[B19] Parker JA Mandleco B Olsen Roper S Freeborn D Dyches TT Religiosity, spirituality, and marital relationships of parents raising a typically developing child or a child with a disability J Fam Nurs 2011 17 1 82 104 21343623 10.1177/1074840710394856

[B20] Havens CA Becoming a resilient family: child disability and the family system Access Today 2005 17 5

[B21] Cuzzocrea F Larcan R Westh F Family and parental functioning in parents of disabled children Nord Psychol 2013 65 3 271 287

[B22] Lach LM Kohen DE Garner RE Brehaut JC Miller AR Klassen AF Rosenbaum PL The health and psychosocial functioning of caregivers of children with neurodevelopmental disorders Disabil Rehabil 2009 31 9 741 752 19736648 10.1080/08916930802354948

[B23] Povee K Roberts L Bourke J Leonard H Family functioning in families with a child with Down syndrome: a mixed methods approach J Intellect Disabil Res 2012 56 10 961 973 22533693 10.1111/j.1365-2788.2012.01561.x

[B24] Coffey JS Parenting a child with chronic illness: a metasynthesis Pediatr Nurs 2006 32 1 51 60 16572539

[B25] Raina P O'Donnell M Schwellnus H Rosenbaum P King G Brehaut J Russell D Swinton M King S Wong M Walter SD Wood E Caregiving process and caregiver burden: conceptual models to guide research and practice BMC Pediatr 2004 4 1 10.1186/1471-2431-4-1 PMC331415 14723791

[B26] Tzoufi M Mantas C Pappa S Kateri M Hyphantis T Pavlou M Mavreas V Siamopoulou-Mavridou A The impact of childhood chronic neurological diseases on Greek families Child Care Health Dev 2005 31 1 109 115 15658971 10.1111/j.1365-2214.2005.00492.x

[B27] Eker L Tüzün EH An evaluation of quality of life of mothers of children with cerebral palsy Disabil Rehabil 2004 26 23 1354 1359 15742980 10.1080/09638280400000187

[B28] Bemister TB Brooks B Kirton A Development, reliability, and validity of the APSP Parental Outcome Measure Pediatr Neurol 2014 51 1 43 52 24938139 10.1016/j.pediatrneurol.2014.01.052

[B29] King S Teplicky R King G Rosenbaum P Family-centered service for children with cerebral palsy and their families: a review of the literature Semin Pediatr Neurol 2004 11 1 78 86 15132256 10.1016/j.spen.2004.01.009

[B30] Kirton A deVeber G Advances in perinatal ischemic stroke Pediatr Neurol 2009 40 3 205 214 19218034 10.1016/j.pediatrneurol.2008.09.018

[B31] Zigmond AS Snaith RP The Hospital Anxiety and Depression Scale Acta Psychiatr Scand 1983 67 6 361 370 6880820 10.1111/j.1600-0447.1983.tb09716.x

[B32] Bjelland I Dahl AA Haug TT Neckelmann D The validity of the Hospital Anxiety and Depression Scale: an updated literature review J Psychosom Res 2002 52 2 69 77 11832252 10.1016/s0022-3999(01)00296-3

[B33] Cohen S Kamarck T Mermelstein R A global measure of perceived stress J Health Soc Behav 1983 24 4 385 396 6668417

[B34] Lessenberry BM Rehfeldt RA Evaluating stress levels of parents of children with disabilities Except Child 2004 70 2 231 244

[B35] Varni JW Sherman SA Burwinkle TM Dickinson PE Dixon P The PedsQL Family Impact Module: preliminary reliability and validity Health Qual Life Outcomes 2004 2 55 15450120 10.1186/1477-7525-2-55 PMC521692

[B36] Panepinto JA Hoffmann RG Pajewski NM A psychometric evaluation of the PedsQL Family Impact Module in parents of children with sickle cell disease Health Qual Life Outcomes 2009 7 1 32 38 19371442 10.1186/1477-7525-7-32 PMC2678996

[B37] Spanier GB Measuring dyadic adjustment: new scales for assessing the quality of marriage and similar dyads J Marriage Fam 1976 38 1 15 28

[B38] Graham JM Liu YJ Jeziorski JL The Dyadic Adjustment Scale: a reliability generalization meta-analysis J Marriage Fam 2006 68 3 701 717

[B39] Schumm WR Characteristics of the Kansas Marital Satisfaction Scale in a sample of 79 married couples Psychol Rep 1983 53 1 583 588

[B40] Schumm WR Paff-Bergen LA Hatch RC Obiorah FC Copeland JM Meens LD Bugaighis MA Concurrent and discriminant validity of the Kansas Marital Satisfaction Scale J Marriage Fam 1986 48 2 381 387

[B41] Sabatelli RM Measurement issues in marital research: a review and critique of contemporary survey instruments J Marriage Fam 1988 50 4 891 915

[B42] de Veber GA MacGregor D Curtis R Mayank S Neurologic outcome in survivors of childhood arterial ischemic stroke and sinovenous thrombosis J Child Neurol 2000 15 5 316 324 10830198 10.1177/088307380001500508

[B43] Bailey DB Golden RN Roberts J Ford A Maternal depression and developmental disability: research critique Ment Retard Dev Disabil Res Rev 2007 13 4 321 329 17979207 10.1002/mrdd.20172

[B44] Swallow V Macfadyen A Santacroce SJ Lambert H Fathers’ contributions to the management of their child’s long-term medical condition: a narrative review of the literature Health Expect 2012 15 2 157 175 21624023 10.1111/j.1369-7625.2011.00674.x PMC5060611

[B45] Statistics Canada Table 111-0009 Median total income, by family type, by province and territory [table] CANSIM [database]. Last updated October 2013. http://www.statcan.gc.ca/tables-tableaux/sum-som/l01/cst01/famil108a-eng.htm (accessed December 1, 2013)

[B46] Rentinck ICM Ketelaar M Schuengel C Stolk J Lindeman E Jongmans MJ Gorter JW Short-term changes in parents’ resolution regarding their young child’s diagnosis of cerebral palsy Child Care Health Dev 2010 36 5 703 708 20412145 10.1111/j.1365-2214.2010.01077.x

[B47] Whittingham K Wee D Sanders MR Boyd R Sorrow, coping and resiliency: parents of children with cerebral palsy share their experiences Disabil Rehabil 2013 35 17 1447 1452 23167453 10.3109/09638288.2012.737081

[B48] Canary HE Creating supportive connections: A decade of research on support for families of children with disabilities Health Commun 2008 23 5 413 426 18850389 10.1080/10410230802342085

[B49] Carona C Crespo C Canavarro M Similarities amid the difference: Caregiving burden and adaptation outcomes in dyads of parents and their children with and without cerebral palsy Res Dev Disabil 2013 34 3 882 893 23291505 10.1016/j.ridd.2012.12.004

[B50] Cohen J Statistical Power Analysis for the Behavioral Sciences 1988 New York: Routledge

[B51] Cohen J A power primer Psychol Bull 1992 112 1 155 19565683 10.1037//0033-2909.112.1.155

