# Supplementary material for: Parent and family impact of raising a child with perinatal stroke
Source: BMC Pediatr. 2014 Jul 14;14:182. doi: 10.1186/1471-2431-14-182 (PMC4107574; doi:10.1186/1471-2431-14-182)
Supplement: Additional file 1 — Demographics questionnaire: parents of children with perinatal stroke. [file 1471-2431-14-182-S1.docx]

Demographics Questionnaire: Parents of Children with Perinatal Stroke

1. How old are you? ___________
2. How old is your child who has a diagnosis of perinatal stroke? ___________
3. What is your child’s sex? ☐ Male ☐ Female
4. Have you been a caregiver for your child with perinatal stroke since his or her birth?

☐ Yes ☐ No

- 1. If **no**, how many years have you been a caregiver to this child? _______________

1. Does your child have impairments? Please check all that apply:

☐ Motor

☐ Cognitive (e.g., learning difficulties)

☐ Language (e.g., speech impediments or language delay)

☐ Behavioural

☐ Visual (e.g., cross-eyed or poor eye sight)

☐ Seizures

☐ Unknown

1. Please rate the severity of your child’s impairments on a scale of **0** (non-existent) to **4** (severe) based on your observations:

- **Please enter 0 if an option is not applicable**

_______ Motor

_______ Cognitive

_______ Language

_______ Behavioural

_______ Visual

_______ Seizures

1. In your opinion, how severe is your child’s condition overall?

☐ Mild ☐ Moderate ☐ Severe

1. How old was your child with you first noticed that he/she may have a neurological concern? ___________
2. How old was your child when he/she received a diagnosis of perinatal stroke? ___________
3. Has your child EVER experienced a seizure?

☐ Yes ☐ No

- 1. If **yes**, is your child currently taking anti-epileptic medication to manage his/her seizures? ☐ Yes ☐ No

1. Is your child the youngest, middle or eldest child in your immediate family?

☐ Youngest

☐ Middle

☐ Oldest

☐ Not applicable (only child)

1. How many kids do you have?

☐ 1

☐ 2

☐ 3

☐ 4+

1. Does anyone else residing in your home have a chronic illness or condition? ☐ Yes ☐ No
   1. If **yes**, please indicate who and which illness or condition he or she has:

_____________________________________________________________________

1. What is your current caregiver status?

☐ Lone caregiver in the home

☐ Co-caregiver in the home (e.g., partner or adult family member also cares for the child)

☐ Other (please specify): __________________________________________

1. What best describes your caregiver status throughout your child’s life up to now?

☐ Lone caregiver in the home

☐ Co-caregiver in the home (e.g., partner or adult family member also cares for the child)

☐ Other (please specify): __________________________________________

1. Do you receive community support for caregiving (e.g., formal respite care)? ☐ Yes ☐ No
   1. If **yes**, please specify: ____________________________________________________
2. Do you receive community support for services (e.g., funding for children with disabilities)?

☐ Yes ☐ No

- 1. If **yes**, please specify: ____________________________________________________

1. On a scale of **0** (not at all) to **4** (extremely), please rate how helpful the following people or resources have been in caring for your child with perinatal stroke:

*** Please enter 0 if an option is not applicable**

| Spouse/partner |  | Friends |  |
| --- | --- | --- | --- |
| Child’s siblings |  | Community supports |  |
| Grandparents |  |  |  |

1. Approximately how many hours a week do you spend caring for your child with perinatal stroke?

☐ <10

☐ 10-20

☐ 20-30

☐ 30-40

☐ >40

1. Approximately how many hours a week do you spend working outside of your home?

☐ <10

☐ 10-20

☐ 20-30

☐ 30-40

☐ >40

1. What is your marital status? ☐ Married/common-law

☐ Divorced/separated – currently single

☐ Divorced/separated – currently remarried/common-law

☐ Single

1. How would you rate your marriage/ relationship prior to having a child with perinatal stroke?

☐ Very satisfying

☐ Satisfying

☐ Neutral

☐ Dissatisfying

☐ Very dissatisfying

☐ Not applicable (I was not in a relationship at the time)

1. Please indicate how having a child with perinatal stroke has impacted your marriage/ relationship:

☐ Strengthened the relationship

☐ Did not have a significant impact on the relationship

☐ Placed strain on the relationship

☐ Not applicable

1. Prior to the birth of your child with perinatal stroke, did you ever feel that you might need to seek psychological services (e.g., for anxiety or depression)? ☐ Yes ☐ No
2. Are you currently seeking psychological services? ☐ Yes ☐ No
3. Are you currently taking any medications for a psychological disorder? ☐ Yes ☐ No
   1. If **yes**, please specify the type of medications: _________________________________

_________________________________________________________________________

1. Do you fluently read and write in English? ☐ Yes ☐ No
2. What is the highest level of schooling you obtained?

☐ Grade school certificate

☐ High school certificate

☐ College certificate or diploma

☐ Bachelor’s degree

☐ Master’s, doctorate, or professional (e.g., law, dentistry, pharmacy) degree

1. Current occupation (if applicable): ______________________________________________
2. What was the total gross income (before taxes end deductions) in your household in the past year? ☐ < $30,000

☐ $30,000 to $70,000

☐ $71,000 to $110,000

☐ $111,000 to $150,000

☐ >$151,000

1. Please indicate your ethnic origin by checking off the appropriate boxes:

|  | |
| --- | --- |
| ☐ | Hispanic/Latino (Mexican, Central and South American) |
| ☐ | Pacific Islander (Australian Aboriginal, Polynesian-Hawaiians, New Zealanders, Tahitians, Samoans, Melanesian, Micronesian) |
| ☐ | Caucasian/White (North American, European, Australian, New Zealand, Former Soviet Union) |
| ☐ | Black (African, African American/Canadian, Caribbean: excluding North Africa) |
| ☐ | Southeast Asian (Chinese, Japanese, Korean, Vietnamese, Cambodian, Thai, Laotian, Taiwanese, Filipino, Malaysian) |
| ☐ | East Indian/South Asian (East Indian, Pakistani, Sri Lankan, Bangladeshi) |
| ☐ | Middle Eastern (North African, Arab Countries) |
| ☐ | First Nations/Aboriginal (Canadian/American) |
| ☐ | Other (please provide country): ________________________________ |
| ☐ | Unknown |

27. Please indicate the ethnic origin of the other biological parent of your child by checking off the appropriate boxes:

|  | |
| --- | --- |
| ☐ | Hispanic/Latino (Mexican, Central and South American) |
| ☐ | Pacific Islander (Australian Aboriginal, Polynesian-Hawaiians, New Zealanders, Tahitians, Samoans, Melanesian, Micronesian) |
| ☐ | Caucasian/White (North American, European, Australian, New Zealand, Former Soviet Union) |
| ☐ | Black (African, African American/Canadian, Caribbean: excluding North Africa) |
| ☐ | Southeast Asian (Chinese, Japanese, Korean, Vietnamese, Cambodian, Thai, Laotian, Taiwanese, Filipino, Malaysian) |
| ☐ | East Indian/South Asian (East Indian, Pakistani, Sri Lankan, Bangladeshi) |
| ☐ | Middle Eastern (North African, Arab Countries) |
| ☐ | First Nations/Aboriginal (Canadian/American) |
| ☐ | Other (please provide country): ________________________________ |
| ☐ | Unknown |

28. Please indicate how much you agree or disagree with the following statements:

|  | **Strongly Agree** | **Slightly Agree** | **Neither Agree Nor Disagree** | **Slightly Disagree** | **Strongly Disagree** |
| --- | --- | --- | --- | --- | --- |
| Professionals at clinic have educated me about my child’s condition |  |  |  |  |  |
| I have discussed my concerns about my child's condition with a professional who specializes in perinatal stroke |  |  |  |  |  |
| I have educated myself about my child's condition through books, the Internet, and other sources |  |  |  |  |  |
| I have reviewed information on the Calgary Pediatric Stroke Program website |  |  |  |  |  |
| I have attended support groups for parents of children with perinatal stroke |  |  |  |  |  |

Thank you for taking the time to complete this questionnaire. Your results will be kept confidential.
